# Identifying precondition configurations of mathematics anxiety among middle school students in China: using NCA and QCA approaches

**DOI:** 10.3389/fpsyg.2024.1329570

**Published:** 2024-09-16

**Authors:** Kai Zhang, Jinhua Zhou

**Affiliations:** ^1^School of Public Administration, Sichuan University, Chengdu, China; ^2^Liupanshui Second Experimental Middle School, Liupanshui, China

**Keywords:** mathematics anxiety, precondition, necessary condition analysis, qualitative comparative analysis, middle school

## Abstract

**Introduction:**

Addressing mathematics anxiety is important to ensure that students achieve good academic performance and maintain their mental health during the critical middle school period. However, previous studies have focused on the separate effects of the preconditions for mathematics anxiety, ignoring the interaction of factors. Therefore, this study aims to identify the determinants of mathematics anxiety from the perspective of complex systems via necessary condition analysis (NCA) and qualitative comparative analysis (QCA). To the best of our knowledge, this is the first study to identify configurations of preconditions of mathematics anxiety among middle school students.

**Methods:**

A total of 183 middle school students aged 16 to 19 years (*M*_age_ = 17.47, SD = 0.89) in China participated in this cross-sectional study. The outcome variable of the study is mathematics anxiety, and the condition variables include mathematics grade, parental support, learning motivation, learning planning, and learning interest.

**Results:**

The necessity condition analysis shows that not all the condition variables constitute the necessity condition of mathematics anxiety alone. Four paths for the influence of multiple condition variables on mathematics anxiety are identified via the configuration analysis. Notably, even students with high mathematics scores and learning interest still experience mathematics anxiety due to a lack of practical parental support and learning motivation. High levels of parental support can exacerbate the mathematics anxiety of students under two conditions: 1) a lack of learning motivation and learning plans, and 2) interest in learning but low mathematics scores and unclear learning plans.

**Discussion:**

This study highlights the need to consider the comprehensive impact of mathematics anxiety, and the findings will help educators and researchers identify the different characteristics of mathematics anxiety in student populations.

## 1 Introduction

As a crucial subject in the middle school curriculum, mathematics involves the study of quantity, structure, change, space, and information. Mathematics is also a vital tool for cultivating students’ logical thinking and problem-solving abilities (Moliner and Alegre, 2022; Passolunghi et al., 2016). With the deepening of the content of mathematics courses, many students have begun to feel stressed and uneasy, resulting in mathematics anxiety. Addressing mathematics anxiety is critical to improving students’ academic performance and mental health. However, the reasons for many students’ mathematics anxiety differ (Li et al., 2021; Madjar et al., 2018; Richardson and Suinn, 1972; Suárez-Pellicioni et al., 2016). Although numerous studies have highlighted the separate effects of prerequisites on mathematics anxiety, there has been a lack of research on their complex interactions. Exploring these prerequisites and their interactions can provide educators with more scientific ways to help students cope effectively with mathematics anxiety.

Mathematics anxiety is regarded as a complex phenomenon caused by many factors. The cognitive and affective aspects of mathematics instruction play a central role in determining its effectiveness and the resulting outcomes for students. Research suggests that active learning strategies, such as problem-based learning, inquiry-based learning, and collaborative learning, can enhance students’ cognitive engagement and understanding of mathematical concepts (e.g., Freeman et al., 2014; Prince, 2004). These approaches encourage students to actively participate in the learning process, which can lead to deeper comprehension and retention of mathematical principles. A positive and supportive learning environment is crucial for promoting students’ affective engagement and reducing mathematics anxiety (Hembree, 1990). Teachers who provide encouragement, support, and constructive feedback can help alleviate students’ anxiety and foster a sense of belonging and confidence in their mathematical abilities. Personalized learning approaches, such as differentiated instruction and adaptive learning technologies, allow students to progress at their own pace and receive targeted support where needed. Encouraging intrinsic motivation in mathematics learning can improve student cognitive and affective engagement and reduce mathematics anxiety (Ryan and Deci, 2000). Activities that emphasize the real-world relevance of mathematical concepts, promote curiosity and exploration, and provide opportunities for autonomy and self-directed learning can foster a sense of intrinsic motivation and enjoyment in mathematics. Given these cognitive and emotional effects, individual differences are critical to optimizing the effectiveness of math instruction.

Individual differences impact the effectiveness of mathematics teaching. Research suggests that individuals have different cognitive styles, such as visual–spatial or verbal–linguistic styles, which can affect how they process mathematical information (Pashler et al., 2008). Tailoring instruction to match students’ cognitive styles can enhance their understanding and retention of mathematical concepts (Pane et al., 2014). Additionally, students may have different learning preferences, such as auditory or visual learning styles. Adapting instructional methods to accommodate these preferences can improve engagement and learning outcomes. In addition, experience with mathematics, including past successes or failures, can shape students’ attitudes and confidence levels. Students with positive experiences may approach math instruction with greater motivation and self-efficacy, whereas those with negative experiences may exhibit mathematics anxiety or avoidance behaviors (Hembree, 1990). In summary, considering individual differences in cognitive styles, learning preferences, and past experiences can inform the design and delivery of math instruction, making it more effective for diverse learners.

A complex systems approach provides a holistic lens through which the multifaceted, intertwined conditions influencing mathematics anxiety can be deciphered. The purpose of this study is to analyze the preparation configurations of mathematics anxiety. Mathematics anxiety is not a product of linear causality but emerges from a web of interconnected, dynamic systems encompassing individual, contextual, and sociocultural dimensions (Rickles et al., 2007; Stella, 2022). Configuration analysis in educational research has heralded nuanced insights into various phenomena, delineating necessary and sufficient conditions for particular outcomes, which has great potential in educational research (Marx et al., 2014). Necessary condition analysis (NCA) focuses on identifying conditions without which the outcome cannot occur (Dul, 2016). Qualitative comparative analysis (QCA) enables the exploration of causes and effects, illuminating how different combinations of conditions can lead to the same outcome (Zschoch, 2011).

Although many studies have explored the various conditions contributing to mathematics anxiety and its multifaceted effects, there is still a gap in how to analyze it within a complex systems framework. Most studies use linear models to analyze the influencing conditions of mathematics anxiety (e.g., Haynes et al., 2004; Rossi et al., 2022). In addition, many studies have focused only on the impact of a single condition on mathematics anxiety (e.g., Radišić et al., 2015), ignoring the interaction and synergy between various conditions. This study stands out because of its analysis from a complex systems perspective, which views mathematics anxiety as a complex system consisting of various interrelated conditions. Moreover, we use QCA and NCA to explore how various conditions combine into different configurations that lead to different levels of mathematics anxiety. Given that having a productive youth population is critical to future social advancement, delving into the roots of mathematics anxiety is critical to pave the way for effective educational strategies and interventions. Additionally, while the symptoms of mathematics anxiety have been studied, the existing research has been somewhat scattered with respect to determining how it works, and related studies have mostly examined complex systems (e.g., Huber and Artemenko, 2021; Stella, 2022; Wang et al., 2015; Zivkovic et al., 2023). A comprehensive mixed-methods approach has the potential to reveal nuanced insights into the multifaceted interplay within the complex relationships.

The main research questions of this study are as follows. In what configuration do preconditions affect middle school students’ mathematics anxiety? Which preconditions are necessary or sufficient conditions to cause mathematics anxiety? Through the analysis of the configuration path of mathematics anxiety, this study will contribute to a deeper understanding of the preconditions of mathematics anxiety and provide a theoretical and practical basis for the development of more effective intervention measures.

### 1.1 Mathematics anxiety in the light of the theoretical framework

To determine where mathematics anxiety comes from, this study uses four important theories: control value theory, social cognitive theory, achievement goal theory, and self-determination theory. These theories aid in our understanding of the potential roles that significant variables such as mathematics grades, parental support, learning motivation, learning planning, and learning interest may play in this phenomenon. The following sections briefly summarize these theoretical stances, highlighting their significance in understanding how these variables interact to shape students’ experiences with mathematical fear.

The control value theory focuses on the origins of emotions, the mechanisms of their effects, and the interactions among various conditions (Pekrun, 2006). The theory posits that students’ expectations of success (influenced by past grades) and subjective appraisal of the task (influenced by interest) are critical determinants of emotional experience. Students who have previously received low mathematics grades, in particular, may have lower self-efficacy views and mistrust their ability to succeed. Furthermore, when a student finds mathematics dull, their view of its utility diminishes, exacerbating their concerns. These findings suggest that a lack of interest in arithmetic combined with poor academic performance may be a powerful combination for increased anxiety.

According to Bandura (2001), social cognitive theory elucidates the impact of parental support on children’s mathematical anxiety. This underscores the importance of social modeling and observational learning (Holmes et al., 2020). Children are more likely to absorb these worries and form similar negative self-perceptions when they observe parental anxiety about mathematics or expressions of doubt about mathematical ability, either for themselves or for their children (Klee et al., 2022). Therefore, essential elements of parental support include showing children how to have a growth mindset, believing in their abilities, and setting a positive example of how to deal with math difficulties.

The achievement goal theory offers useful insights into the roles of learning planning and motivation (Ames, 1992). This paradigm highlights the importance of goal orientation in shaping students’ emotional responses and behavioral tendencies. Ruzek et al. (2016) reported that students with mastery approach goals, which involve a strong desire to learn and an emphasis on competence, are more motivated to study and employ more effective learning practices. These students also tend to engage in more effective learning planning, which contributes to their feelings of control and reduced anxiety. These students also tend to engage in more effective learning planning, which contributes to their feelings of control and reduced anxiety. Conversely, people with a performance-avoidance focus, which is primarily concerned with avoiding negative evaluations of their abilities, may experience increased anxiety as a result of their mistrust of their learning strategies and fear of failure.

Self-determination theory complements achievement goal theory by highlighting how different sources of motivation can influence students’ experiences (Deci and Ryan, 2000). When students have a genuine desire to learn, and value mastering mathematics for their own purpose, they are more likely to participate in independent and persistent learning, resulting in significantly lower levels of anxiety (Sutter-Brandenberger et al., 2018). Parental support, however, might undermine a student’s sense of autonomy when it takes the form of strict learning practices or pressure to earn excellent mathematics grades. This can tip the motivational balance in favor of extrinsic demands, potentially increasing anxiety.

Mathematics anxiety is often associated with reduced self-efficacy, diminished perceived value of math, social observational learning, achievement goal orientation, and intrinsic and extrinsic motivation (Ames, 1992; Deci and Ryan, 2000; Holmes et al., 2020; Klee et al., 2022; Pekrun, 2006; Ruzek et al., 2016; Sutter-Brandenberger et al., 2018). Taking these conditions collectively can assist in comprehending and resolving mathematics anxiety, providing a crucial direction to improve students’ math learning experiences and performance.

### 1.2 Key conditions influencing mathematics anxiety

A theoretical framework was used to categorize and analyze the five necessary prerequisites: math achievement, parental support, learning motivation, learning plans, and learning interest. This leads to a better understanding of the complex interplay of factors that contribute to mathematics anxiety in middle school students. The conditions are not only considered individually but are also analyzed by considering the configuration of the interactions of the factors highlighted in the theoretical model. This approach acknowledges that different combinations of these preconditions can lead to higher levels of anxiety.

#### 1.2.1 Student performance and engagement

This category encompasses factors directly related to a student’s academic performance and their engagement with mathematics.

##### 1.2.1.1 Mathematics grade

Past performance in mathematics, as reflected in grades, can significantly influence a student’s confidence and anxiety levels. Lower grades may contribute to negative self-perceptions and a fear of future failure, potentially leading to heightened anxiety (Ashcraft and Moore, 2009; Ma and Xu, 2004). This study proposes the following hypothesis on the basis of the findings:

*H1*: Mathematics grades have a significant negative effect on mathematics anxiety.

##### 1.2.1.2 Learning interest

A genuine interest in, and enjoyment of, mathematics can serve as a protective factor against anxiety. Students who are intrinsically motivated to learn and explore mathematical concepts are less likely to experience anxiety, even when faced with challenges (Krapp and Prenzel, 2011). On the basis of this information, this study presents the following hypothesis:

*H2*: Learning interest has a significant negative effect on mathematics anxiety.

#### 1.2.2 Parental and home environment

The home environment, particularly the nature of parental support, can play a critical role in shaping a child’s attitudes toward mathematics and their vulnerability to anxiety.

##### 1.2.2.1 Parental support

Positive and supportive parental involvement, characterized by encouragement, positive reinforcement, and a growth mindset, can create a more secure and conducive learning environment, reducing mathematics anxiety (Kreslins et al., 2015; Maloney et al., 2015). Therefore, this study proposes the following hypotheses:

*H3*: Parental support has a significant negative effect on mathematics anxiety.

#### 1.2.3 Learning strategies and motivation

This category focuses on the strategies that students employ and their underlying motivations for learning mathematics.

##### 1.2.3.1 Learning motivation

Intrinsic motivation to learn and master mathematical concepts has been associated with lower levels of mathematics anxiety (Middleton and Spanias, 1999; Wigfield and Meece, 1988). Students driven by intrinsic motivation view challenges as opportunities for growth, fostering resilience and a more adaptive response to difficulties. This study proposes the following hypothesis:

*H4*: Learning motivation has a significant negative effect on mathematics anxiety.

##### 1.2.3.2 Learning planning

Effective learning strategies, including planning, organization, and self-regulation, can help students manage their learning process, breakdown complex tasks, and reduce the feeling of being overwhelmed, thus mitigating anxiety (Pajares and Miller, 1994). In light of this, the study proposes the following hypothesis:

*H5*: Learning planning has a significant negative effect on mathematics anxiety.

In addition, this study acknowledges that these factors are not independent of a configurational perspective. We recognize that students may encounter varying degrees of anxiety depending on their mathematics grades, the level of parental support they receive, their intrinsic and extrinsic motivations for learning, their ability to plan and organize their learning, and their genuine interest in mathematics. On this basis, we propose the following hypothesis:

*H6*: The precondition for mathematical anxiety is the combination action, and the configuration path is not unique.

**Figure 1:**
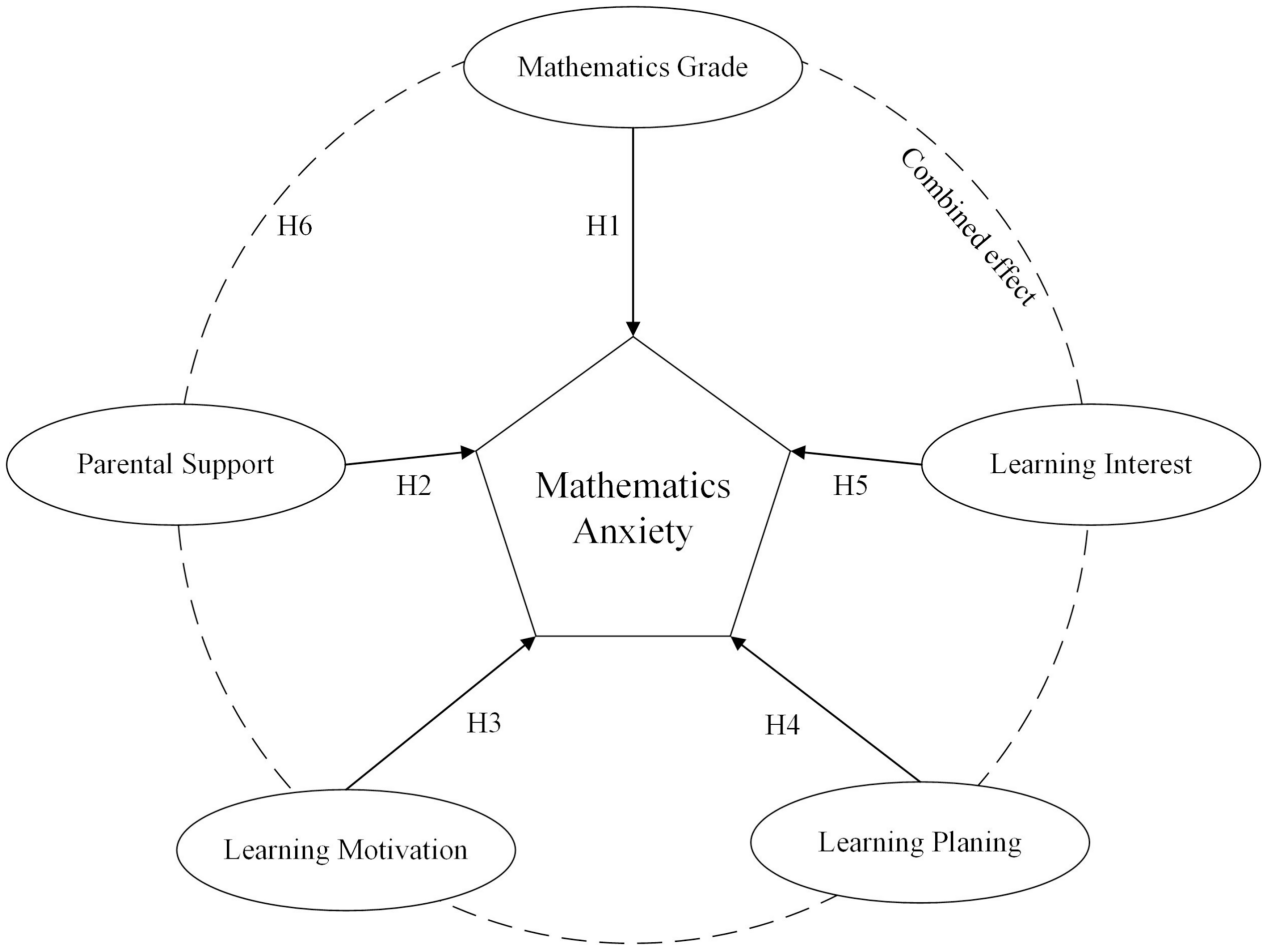
Configuration path of mathematics anxiety.

Figure 1 provides a visual representation of how these five key preconditions may dynamically interact to shape students’ general level of mathematics anxiety. By examining these intricate relationships, this study aims to provide a more comprehensive understanding of mathematics anxiety, and identifies specific combinations of preconditions that contribute most significantly to its emergence in Chinese middle school students.

## 2 Materials and methods

### 2.1 Participants

The main participants in the poll were students from five middle schools in Liupanshui city with ages ranges from 16 to 19. We implemented a field investigation to guarantee the success of the survey among the intended population. The survey focused on gathering information on the respondents’ demographics, mathematics grades, mathematics anxiety, and learning situation. We performed two rounds of data collection in February and June 2023. During the initial phase of the survey, we delivered a total of 23 questionnaires as a preliminary measure to refine and improve the questionnaire design. However, the later analysis of the results did not include these questionnaires. In the second round, we collected a total of 193 questionnaires, 183 of which were genuine, accounting for 94.82% of the total. Each participant voluntarily took the survey. The number of students used for analysis was 183 (*M*_age_ = 17.47, SD = 0.89). Most (53.01%) were girls: 1st grade (34.97%), 2nd grade (31.55%), and 3rd grade (33.88%).

### 2.2 Instruments

The Mathematics Anxiety Scale (MAS) was created to gather data pertaining to students’ anxiety toward mathematics. The measure was developed with questions derived from the Abbreviated Mathematics Anxiety Scale (AMAS) (Hopko et al., 2003), as suggested by experts in the field. The scale consists of 16 items and follows the attributes of the 5-item Likert scale. Each item is rated on a scale from no anxiety (1) to very anxious (5). The condition variables consisted of five parts: mathematics grade (1 item), parental support (2 items), learning motivation (3 items), learning plans (2 items), and learning interest (2 items). Each item ranges from particularly nonconforming (1) to particularly conforming (5). We obtained the sum score for the total MAS score by adding the scores of all 16 items, which ranged from 16 to 80. The Mathematics anxiety questionnaire is shown in the Supplementary Appendix.

### 2.3 Data analysis

**Figure 2:**
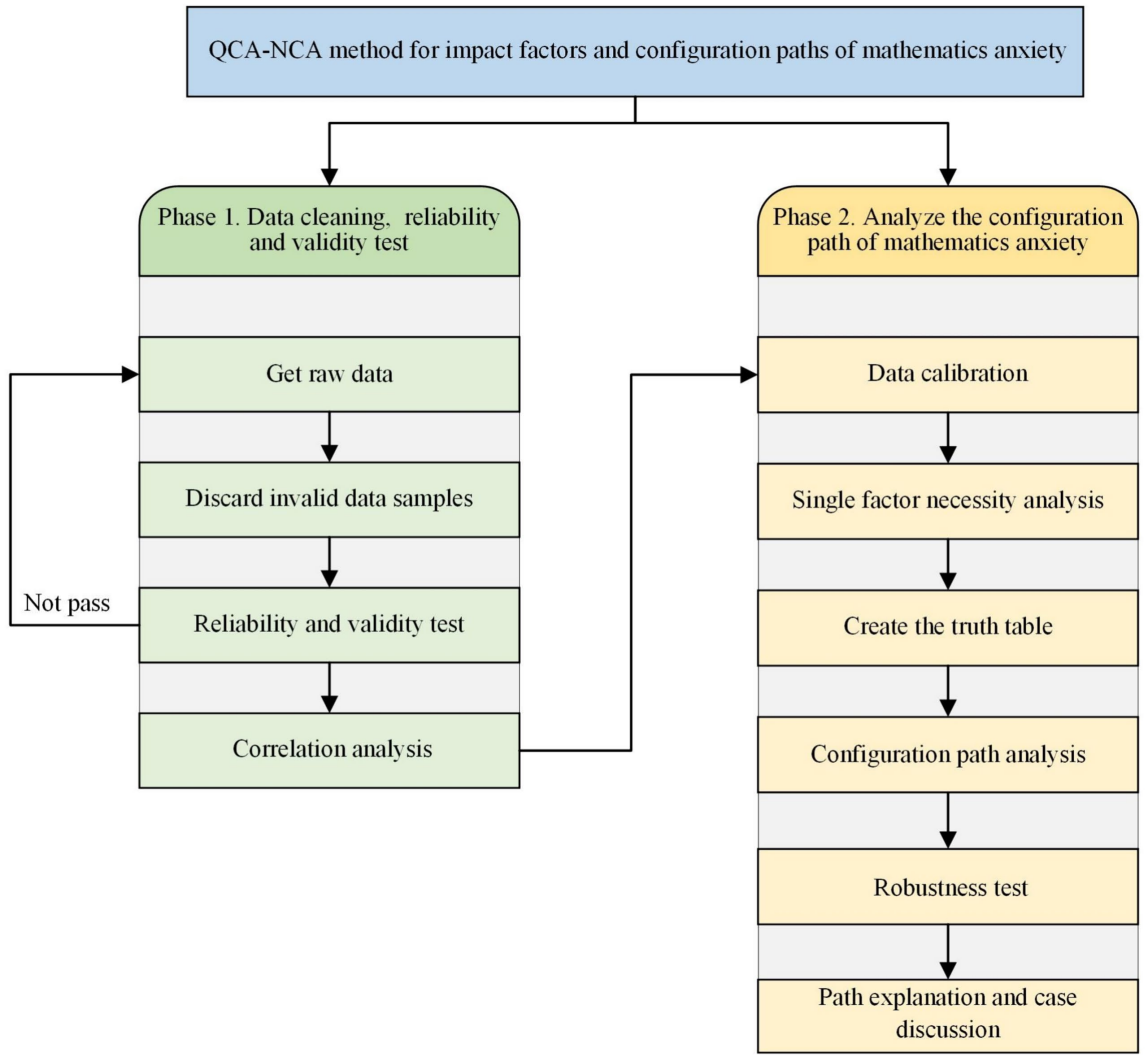
The application framework of the study method.

This section describes the analytical procedures used to investigate the different mechanisms through which preconditions affect mathematics anxiety. Initially, we prioritized the accuracy and reliability of our data and scrutinized the psychometric characteristics of our measurements via *SPSS 26.0*. We subsequently performed correlational analyses to investigate the associations between variables. Moreover, we employed necessary condition analysis (NCA) in *R 3.3.1* to ascertain any indispensable requirements for mathematics anxiety. NCA elucidates whether a particular condition is a requisite for an outcome, meaning that the outcome cannot occur without it, even if it is insufficient to cause the outcome alone (Dul, 2016). The use of fuzzy-set qualitative comparative analysis (fsQCA) in *FSQCA 4.1* ultimately facilitated the identification of specific combinations of preconditions that are associated with increased levels of mathematics anxiety. QCA ascertains how configurations of different conditions can equivalently result in the observed outcome, encapsulating both the complexity and equivocality inherent in social phenomena (Marx et al., 2014). Figure 2 shows the application framework of the study method.

Table 1 presents the test results for the reliability and validity of the mathematics anxiety and condition variables. We conducted an assessment of the questionnaire’s reliability and validity. In terms of reliability, the condition variables’ Cronbach’s α was greater than 0.729, and the Cronbach’s α of mathematics anxiety was 0.984. The findings demonstrated a strong level of internal consistency among the indicators, as reported by Christmann and Van Aelst (2006). The condition variables had an overall Kaiser–Meyer–Olkin (KMO) validity of 0.724, indicating that they were eligible for factor analysis (Bentler and Weeks, 1980). We simultaneously performed a confirmatory factor analysis on both the variables of mathematics anxiety and condition. The standardized load coefficient of each indicator exceeded 0.669. The average variance extraction (AVE) for each variable exceeded 0.577, and the composite reliability (CR) exceeded 0.731, demonstrating efficient extraction of the measurement index within the given situation (Cheung et al., 2023). In addition, we estimated all the variables via the confirmatory factor analysis model. In this model, all latent factors were freely estimated to correlate, allowing exploration of potential relationships among the underlying constructs. The results showed good data fit [*_x_*^2^(265) = 610.87, *p* < 0.001, RMSEA = 0.05, CFI = 0.93, TLI = 0.94, SRMR = 0.03] (Hu and Bentler, 1999). In conclusion, middle school students can assess mathematics anxiety effectively via a questionnaire.

**Table 1:** Results of the reliability and validity tests (*N* = 183).

Variable	Indicator	Loading	Cronbach’s α	AVE	CR
Mathematics anxiety	MA1	0.951	0.984	0.805	0.985
	MA2	0.947			
	MA3	0.893			
	MA4	0.934			
	MA5	0.951			
	MA6	0.956			
	MA7	0.941			
	MA8	0.906			
	MA9	0.891			
	MA10	0.922			
	MA11	0.926			
	MA12	0.905			
	MA13	0.729			
	MA14	0.669			
	MA15	0.781			
	MA16	0.913			
Parental support	PS1	0.925	0.826	0.711	0.831
	PS2	0.761			
Learning motivation	LM1	0.774	0.806	0.581	0.806
	LM2	0.807			
	LM3	0.702			
Learning planning	LP1	0.760	0.744	0.595	0.746
	LP2	0.781			
Learning interest	LI1	0.770	0.729	0.577	0.731
	LI2	0.747			

The mathematics grade is directly input into the data and does not need to be measured, and the reliability and validity of the scale are not included. MA, mathematics anxiety; MG, mathematics grade; PS, parental support; LM, learning motivation; LP, learning planning; LI, learning interest. The following abbreviations remain consistent.

The QCA method requires that the number of cases and the number of condition variables match to avoid the limited diversity problem that often occurs in research. In theory, as the number of condition variables increases, the number of possible combinations of condition variables increases exponentially, and there are *_k_*^2^ combinations of *_k_* condition variables (Greckhamer et al., 2008). Since there are a total of 5 condition variables in this study, 32 combinations are theoretically generated, so it is reasonable to select 183 students as the sample. FsQCA is an extended and improved version of the QCA method, and introduces fuzzy-set theory to handle part of the collection problem (Zadeh, 1965).

The general operation process of fsQCA is as follows: (1) Calibration. Calibration to involves attributing condition variables to a particular membership set. The subset relationship analysis of necessity and adequacy may be continued only if the original case data are calibrated to set a membership score. (2) Single-condition necessity analysis. Without this condition, the result cannot be produced, meaning that this condition always exists when the result exists. The primary objective is to evaluate the subset relationship between the outcome variable and the condition variables. (3) Create the truth table. The appropriate threshold values are set to screen the data. (4) Analysis of the configuration path. Simple and intermediate solution results are attained to determine the core and edge conditions that are more critical to a particular configuration.

## 3 Results

### 3.1 Correlation analysis

The correlation coefficients between mathematics anxiety and the condition variables are shown in Table 2, which are calculated by the sum scores of the observed indicators for each variable. The Pearson correlation coefficient matrix reveals a significant negative correlation between mathematics anxiety and mathematics grade, learning motivation, learning planning, and learning interest. Notably, these findings support Hypotheses H1, H2, H4, and H5. These results are consistent with those of previous studies (Ashcraft and Moore, 2009; Krapp and Prenzel, 2011; Ma and Xu, 2004; Middleton and Spanias, 1999; Pajares and Miller, 1994; Wigfield and Meece, 1988). Parental support is not correlated with mathematics anxiety; H3 remains unsupported; further configuration analysis is needed to confirm the relationship between mathematics anxiety and parental support.

**Table 2:** Correlation analysis between mathematics anxiety and condition variables (*N* = 183).

	MA	MG	PS	LM	LP	LI
MA	1.000					
MG	−0.133*	1.000				
PS	0.116	−0.089	1.000			
LM	−0.321***	−0.012	0.290***	1.000		
LP	−0.405***	−0.120	0.112	0.532***	1.000	
LI	−0.189***	0.036	0.211***	0.203***	0.333***	1.000

***, **, and *Represent significance levels of 0.1, 1, and 5%, respectively.

### 3.2 Calibration

Before a fsQCA analysis can be performed, the raw data for the outcome and condition variables must be calibrated so that their values are within an acceptable range of 0 to 1. The direct correction method is used to remove abnormal data and improve the accuracy of the analysis results. Fuzzy processing was used to transform each research variable into a fuzzy set, as shown in Table 3. A total of 90, 50, and 10% of the outcome variable and condition variable scores are used as three calibration anchors for full membership, crosspoint, and complete non-membership degrees, respectively.

**Table 3:** Variable calibration anchor settings (*N* = 183).

Variable		Calibration			Mean	SD	Min	Max
		90%	50%	10%				
Outcome	MA	73	48	16	45.21	21.27	16	80
Condition	MG	109.8	59	34	64.31	28.45	12.5	147
	PS	8	3	2	4.16	2.43	2	10
	LM	12	9	3	8.37	3.30	2	15
	LP	8	6	3	5.58	1.90	2	9
	LI	8	6	2	5.57	2.03	2	10

### 3.3 Single-condition necessity analysis

Before performing a configuration analysis, we conduct a single-condition necessity analysis via QCA to ascertain the extent and explanatory power of all the condition variables for the outcome. Generally, we can use consistency and coverage as metrics. Consistency refers to the degree to which specific conditions or combinations of conditions in the research share the existence of specific results in all situations. One can argue that the condition variable serves as the adequacy condition for the outcome variable if its consistency is more significant than 0.8, and it becomes the necessary condition for the outcome variable if the consistency exceeds 0.9 (Ragin, 2006). When its value decreases to 0, the explanatory power is weaker. Coverage refers to the explanatory power of specific conditions or combinations of conditions for a given result. When the value approaches 1, the explanation’s force is greater.

The following are the calculations for consistency and coverage (Ragin, 2006):


(1) $$\begin{array}{l} \text{Consistency}\left(x_{i}\leq y_{i}\right)=\frac{\sum \min\left(x_{i},y_{i}\right)}{\sum x_{i}}\\ \text{Coverage}\left(x_{i}\leq y_{i}\right)=\frac{\sum \min\left(x_{i},y_{i}\right)}{\sum y_{i}} \end{array}$$

According to Table 4, the single condition necessity analysis does not have a consistency greater than 0.9, which means that none of them can independently account for the high level of mathematics anxiety. High mathematics anxiety is the result of a combination of condition variables.

**Table 4:** The necessary condition analysis results of the single condition.

Condition variable	Consistency	Coverage
MG	0.497	0.528
~MG	0.665	0.644
PS	0.617	0.637
~PS	0.565	0.562
LM	0.515	0.523
~LM	0.704	0.712
LP	0.428	0.466
~LP	0.763	0.723
LI	0.606	0.643
~LI	0.624	0.604

~Indicates not using the logical operation. MA, mathematics anxiety; MG, mathematics grade; PS, parental support; LM, learning motivation; LP, learning planning; LI, learning interest. The abbreviations below remain consistent.

**Figure 3:**
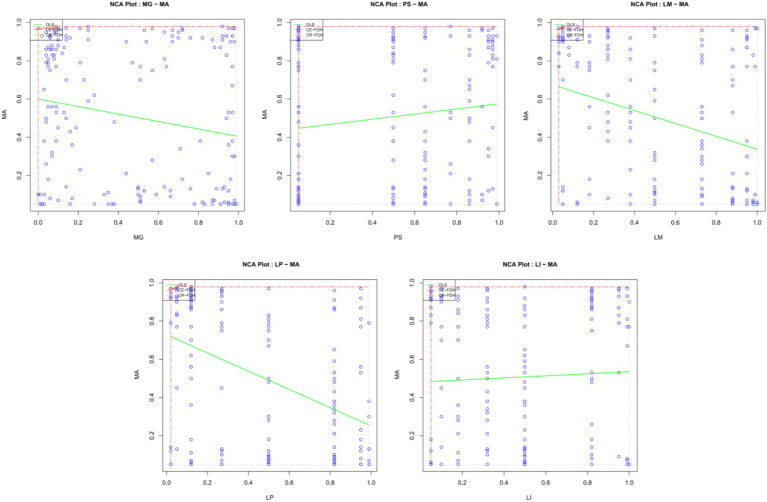
Scatter plot of the NCA analysis results. The blue dots represent observations. The red line is the upper limit of the step function, the ceiling-envelopment-free disposal hull (CE-FDH). The yellow line is the upper limit of the ceiling regression-free disposal hull (CR-FDH). The green line is the ordinary least squares OLS regression line.

Table 5 shows the results of the ceiling regression (CR) and ceiling-envelopment (CE) methods, which are all 100% accurate. The criteria were that the effect size was greater than 0.1 and *p* < 0.05 (Dul et al., 2020), so the results showed that the necessary conditions for mathematical anxiety did not exist. Figure 3 shows the scatter plot of the condition variables based on an NCA analysis. Students with lower math scores, or less clear learning motives or plans, exhibit more severe mathematics anxiety. The higher the level of parental support, the more serious the students’ mathematics anxiety.

**Table 5:** Results of the necessity analysis of the NCA.

Condition variable	Method	C-accuracy	Ceiling zone	Effect	*d*	*p*-value
MG	CR	100	0.000	0.92	0.000	0.894
	CE	100	0.000	0.92	0.000	0.886
PS	CR	100	0.000	0.87	0.000	1.000
	CE	100	0.000	0.87	0.000	1.000
LM	CR	100	0.000	0.90	0.000	0.895
	CE	100	0.000	0.90	0.000	0.895
LP	CR	100	0.000	0.90	0.000	0.829
	CE	100	0.000	0.90	0.000	0.829
LI	CR	100	0.000	0.88	0.000	1.000
	CE	100	0.000	0.88	0.000	1.000

CE is the ceiling-envelopment-free disposal hull (CE-FDH). CR is the ceiling regression-free disposal hull (CR-FDH).

### 3.4 Creating the truth table

The truth table should have 2^5^ different combinations of criteria. Generally, when the raw consistency is significantly greater than 0.8 and the PRI consistency is greater than 0.7, the value of mathematics anxiety is 1; otherwise, the value of mathematics anxiety is 0 (Fiss, 2011). Given the medium-sized sample size of this study, we set the RAW consistency at 0.8 and the PRI consistency at 0.6. After consistency checks and threshold setting, Table 6 shows that seven combinations of conditioning variables lead to high levels of mathematics anxiety.

**Table 6:** Conditional combination truth table (outcome variable = 1).

MG	PS	LM	LP	LI	Number	MA	RAW consist	PRI consist
0	1	0	0	1	3	1	0.927	0.769
0	1	0	0	0	14	1	0.892	0.765
0	0	0	0	1	3	1	0.903	0.744
0	0	0	0	0	19	1	0.860	0.740
1	0	0	0	1	13	1	0.862	0.716
1	1	0	0	0	6	1	0.856	0.710
1	1	0	0	1	2	1	0.890	0.672
0	1	1	1	1	4	1	0.859	0.657

### 3.5 Configuration path analysis

The fsQCA configuration analysis generates complex, intermediate, and parsimonious solutions by eliminating situations that do not meet the frequency and consistency thresholds. To analyze the path combination of high mathematics anxiety more objectively, we combine the intermediate and parsimonious solutions in its path combination analysis. The intermediate solution classifies the configuration of the condition variables, whereas the parsimonious solution distinguishes the condition variables, which include the core and edge conditions. Table 7 displays the results of a path combination analysis that includes condition variables.

**Table 7:** Results of the conditional configuration analysis.

Variable	Configuration path			
	1	2	3	4
MG	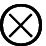		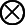	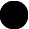
PS		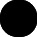	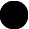	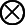
LM	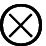	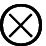		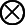
LP	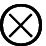	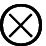	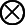	
LI			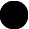	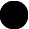
Form	~MG* ~ LM* ~ LP	PS* ~ LM* ~ LP	~MG*PS* ~ LP*LI	MG* ~ PS* ~ LM*LI
Consistency	0.866	0.886	0.874	0.819
Raw coverage	0.423	0.364	0.252	0.231
Unique coverage	0.114	0.079	0.058	0.071
Consistency of solution	0.837			
Coverage of solution	0.641			

Intermediate solution and parsimonious solution. The symbol 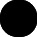 indicates that the core condition variable exists; the symbol 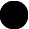 indicates that the edge condition variable exists; blank space indicates that the condition variable is optional; the symbol 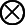 indicates that the edge condition variable does not exist; and the symbol 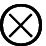 indicates that the core condition variable does not exist. * Indicates that different conditions exist simultaneously; ~ indicates that the condition does not exist. The article follows this standard.

Single-condition necessity analysis and configuration path analysis provide insights into the combined effects of different condition variables on mathematics anxiety. No single condition variable emerges as a necessary determinant of mathematics anxiety. These findings align with Hypothesis H6 that mathematics anxiety is likely influenced by a combination of conditions rather than a single determinant, underscoring the complexity of this phenomenon.

According to Table 5, there are four alternative configurations for mathematics anxiety, and each has a consistency score that is more significant than 0.8. According to the coverage rate of the solution, which is 0.641, the result can explain the composition of mathematics anxiety with a degree of 64.1% and has good explanatory power (Glaesser and Cooper, 2014). This reflects that all configuration paths are sufficient conditions for developing mathematics anxiety. This study presents and discusses four configuration paths for mathematics anxiety based on the distribution of the framework’s core condition variables and edge condition variables.

### 3.6 Robustness test

To examine the viability of the antecedent circumstances for high mathematics anxiety, we increased the thresholds and the consistency thresholds in this study. We increase the consistency criterion from 0.8 to 0.85, and the case-setting threshold from 1 to 3. Table 8 displays the outcomes of the robustness tests. The combination of configuration paths remained the same; the consistency of the solution was 0.849, and the coverage rate was 0.614. The raw coverage, unique coverage, and path consistency did not differ much from the first result and were all within the allowed error range (Glaesser and Cooper, 2014; Ragin, 2006). As a result, the state analysis findings in this study group are robust.

**Table 8:** Results of the robustness test.

Consistency and coverage	Configuration path			
	1	2	3	4
Consistency	0.866	0.882	0.868	0.874
Raw coverage	0.423	0.305	0.276	0.252
Unique coverage	0.098	0.063	0.065	0.058
Consistency of solution	0.849			
Coverage of solution	0.614			

## 4 Discussion

This study takes a group of Chinese middle school students as its research object, analyzes the influencing conditions of mathematics anxiety from the perspective of complex systems, and verifies the configuration path of mathematics anxiety analysis via the NCA and QCA methods. Mathematics grade, parental support, learning motivation, learning planning, and learning interest are selected as the condition variables, and mathematics anxiety is selected as the outcome variable. On the basis of the necessity analysis results of the mixed method, it is found that all the condition variables do not constitute the necessary conditions of mathematics anxiety, indicating that mathematics anxiety is not directly affected by a single condition.

Path 1 indicates that low mathematics grades, unclear learning motivation and learning plans cause high mathematics anxiety. This finding aligns with our hypothesis that poor math performance is directly associated with mathematics anxiety (H1), a result that is consistent with previous research (Ashcraft and Moore, 2009; Geary et al., 2021; Gunderson et al., 2012). Students who lack motivation may struggle to overcome math difficulties, leading to increased anxiety (Guo and Liao, 2022). A practical study plan can help students better manage their study time and resources, thus increasing their sense of control over their math learning. Furthermore, a lack of learning plans can cause students to feel overwhelmed in the face of math challenges, which can exacerbate mathematics anxiety (Mononen et al., 2022). As a result, this path not only supports our hypothesis but also emphasizes the importance of motivation and planning in reducing mathematics anxiety. To better help students, educators and parents should pay attention to the interaction of these conditions and take comprehensive measures to improve their math confidence, stimulate their learning motivation, and help them develop effective learning plans.

Path 2 indicates a stimulating combination of unclear learning motivation, unclear learning planning, and high parental support. This finding is consistent with our Hypothesis H3, which suggests that parental support has a significant negative effect on mathematics anxiety. Students may have increased mathematics anxiety when they have high parental support but low motivation and inefficient learning plans (H4, H5) (Molden and Dweck, 2006; Pajares and Miller, 1994). Instead of having an innate interest in mathematics, students may view parental pressure as their main source of motivation, which might cause anxiety (Middleton and Spanias, 1999). In this case, students may think that their primary reason for studying math is the satisfaction of their parents rather than their own interests or desires (Gunzenhauser et al., 2021). The combination of this misunderstanding and the high expectations of parents may intensify students’ mathematical anxiety (Trigueros et al., 2020). This path not only corroborates our theory but also underscores the significance of taking into account external as well as internal variables when resolving mathematics fear among students. To enhance student performance, instructors and parents must provide assistance and guarantee that students have an intrinsic drive and suitable learning methodologies.

Path 3 indicates a stimulating combination of low mathematics grade, high parental support, unclear learning planning, and high learning interest. This finding supports our Hypothesis H6, which states that the conditions that contribute to mathematics anxiety are diverse, and that the configuration path is not unique. Despite their high interest in mathematics and parental support, children may nonetheless experience mathematics anxiety as a result of low grades and inefficient learning programs (Dou and Zwolak, 2019). Although students are very interested in mathematics and have excellent parental support, a lack of learning plans may impede their growth. An effective study schedule can help students maximize their learning potential and enhance their math skills. As a result, Path 3 not only supports our hypothesis about the interaction of various conditions in forming mathematics anxiety but also emphasizes the importance of taking into account the nuanced relationship between parental support, learning plans, and learning interest when effectively addressing mathematics anxiety. To support students, educators and parents must evaluate these factors and work to establish an environment that encourages them to develop their interests and potential while offering vital learning methodologies and program recommendations (Reinhold et al., 2021; Teppo et al., 2021).

Path 4 indicates high mathematics grades, low parental support, unclear learning motivation, and high learning interest, revealing a unique pattern. This configuration challenges conventional wisdom by demonstrating that even students with high math scores may experience mathematics anxiety under certain circumstances. Despite their academic success, students may still feel anxious about math, particularly if they lack parental support and motivation (Rubach and Bonanati, 2023). The absence of external encouragement and internal drive can lead to wavering attitudes toward math, despite a strong interest in learning (Ahmed et al., 2010; Gunderson et al., 2018). This path helps us reflect on the complex effects of mathematics anxiety, where multiple conditions may be combined or nonlinear. To fully understand and support students, we must consider these conditions across multiple dimensions and ensure that students have the right learning environment and support.

The results of this study not only align with the literature on the relationships between mathematics anxiety, mathematics grade, learning motivation, planning, and interest (Ashcraft and Moore, 2009; Geary et al., 2021; Gunderson et al., 2018; Pashler et al., 2008) but also shed light on the nuanced role of parental support in mitigating mathematics anxiety. While previous research has focused primarily on the positive effects of parental support, this study emphasizes the importance of aligning parental support with students’ individual learning strategies and motivations for effective reduction. Moreover, this study advocates the adoption of a complex systems perspective to comprehend mathematics anxiety, a viewpoint uncommon in the current literature. This perspective highlights the interconnectedness of various conditions influencing mathematics anxiety, providing a theoretical foundation for designing more comprehensive and targeted interventions.

These findings have consequences for educational practices. Mitigating mathematics anxiety requires a holistic approach that focuses on student achievement by addressing learning attitudes, techniques, and motivations, as well as cultural differences in learning and teaching. Mathematics anxiety is influenced by cultural differences in ideas and attitudes, which can affect self-concept and self-efficacy when learning arithmetic concepts (Brown et al., 2020). Students frequently perceive math as a topic that demands memorization rather than comprehending practical applications, and there is a link between this impression and varying levels of mathematics anxiety (Grenier et al., 2020; Lau et al., 2022). Cultivating a positive attitude toward mathematics is essential for creating an environment in which students from various backgrounds feel confident, engaged, and driven to learn. Giving students effective learning practices that align with their cultural learning preferences strengthens their problem-solving abilities and promotes a sense of competence. Adapting parental support to each student’s unique learning methods, goals, and cultural backgrounds is crucial. A partnership between parents and educators to recognize and respond to these specific demands within a culturally sensitive framework can greatly reduce mathematics anxiety.

Applying a comprehensive approach to understanding mathematics anxiety from a complex systems perspective has the potential to impact educational practice. Understanding the collective impact of the many factors that contribute to mathematics anxiety might effectively inform solutions aimed at addressing this problem. We recommend that educators and administrators consider the complex interplay between variables such as learning motivation, attitudes, technology, and family support when developing educational programs.

This study has several limitations: (1) This study uses a group of Chinese middle school children as its research object, and factors such as culture, the education system, and social background may influence the findings. These rules may not be applicable to middle school students in different countries and areas. (2) Although we considered variables such as mathematics grade, parental support, learning motivation, learning planning, and learning interest, we overlooked additional variables such as teacher support, peer relationships, and the school environment that could also influence mathematics anxiety. (3) There could be a reciprocal causal connection between mathematics anxiety and the condition variables, but the NCA and QCA approaches are ineffective in identifying the associations between these variables. (4) Although the NCA and QCA approaches offer valuable insights into the configuration paths of mathematics anxiety, this methodology is also limited by potential difficulties in handling larger datasets or more condition factors. Despite these limitations, this study is important for understanding and addressing mathematics anxiety. Subsequent research can enhance the study’s design, broaden the range of participants, and investigate additional factors that may more fully elucidate the underlying causes and potential interventions for mathematics anxiety.

## 5 Conclusion

This study innovatively uses a mixed approach from a complex systems perspective to analyze the preconditions of mathematics anxiety, which differs from previous studies. The multicondition configuration affects mathematics anxiety, and factors such as mathematics grade, parental support, learning motivation, learning planning, and learning interest do not constitute necessary conditions for mathematics anxiety. Moreover, there are four configuration paths for mathematics anxiety, each presenting different characteristics. Therefore, we should implement targeted programs to alleviate the mathematics anxiety of middle school students and foster their healthy development. Future research will use time series data to analyze how mathematics anxiety changes under complex systems, which will help in deeply analyzing the internal logic of mathematics anxiety.

## Data Availability

The original contributions presented in the study are included in the article/Supplementary materials, further inquiries can be directed to the corresponding authors.
