# Supplementary material for: Identifying precondition configurations of mathematics anxiety among middle school students in China: using NCA and QCA approaches
Source: Front Psychol. 2024 Sep 16;15:1329570. doi: 10.3389/fpsyg.2024.1329570 (PMC11439787; doi:10.3389/fpsyg.2024.1329570)
Supplement: Supplementary file 1 [file Data_Sheet_1.pdf]

Questionnaire

Personal Information

1. What is your gender?

☐Men

☐Women

2. Age: _____. (Gap filling)

3. What grade are you in?

☐ 1st grade

☐ 2nd grade

☐ 3rd grade

4. Last final exam in mathematics: _____. (Fill in the blank)

Mathematics Anxiety

	1	2	3	4	5
1. Prepare for a math test.					
2. Take mid-term and final math exams.					
3. Take out your math textbook and do your homework.					
4. The teacher assigned a lot of difficult math problems and asked them to be finished before the next class.					
5. Think about your math test the week before.					
6. Think about your math test the day before.					
7. Think about your math test an hour before it.					
8. Discovering that you have to take a certain number of math classes to reach a certain goal.					
9. Pick up your math textbook and read a difficult chapter.					
10. When you get your math test score.					
11. Open your math or statistics textbook and see a whole page of math problems.					
12. Math teacher pop quiz.					
13. To calculate a group of addition and subtraction operations, you need to write.					
14. Sign up for a math class.					
15. Listen to students explain mathematical formulas.					
16. See complex mathematical graphs and formulas.					

Note: no anxiety (1) to very anxious (5).

Parental support

1. Your parents take time out to help you with math.

☐Very inconsistent

☐More consistent

☐Generally consistent

☐More consistent

☐Fully consistent

2. Your parents enroll you in after-school math classes every semester.

☐Very inconsistent

☐More consistent

☐Generally consistent

☐More consistent

☐Fully consistent

Learning motivation

1. I like math tests very much if I don’t care about ranking.

☐Very inconsistent
☐More consistent
☐Generally consistent
☐More consistent
☐Fully consistent

2. You don’t need to be prodded to learn math.

☐Very inconsistent
☐More consistent
☐Generally consistent
☐More consistent
☐Fully consistent

3. When you encounter a math problem that you can’t understand, you do everything you can to figure it out.

☐Very inconsistent
☐More consistent
☐Generally consistent
☐More consistent
☐Fully consistent

Learning planning

1. The amount of time you are able to arrange for study and recreation.

☐Very inconsistent
☐More consistent
☐Generally consistent
☐More consistent
☐Fully consistent

2. You have a study plan for each semester or month.

☐Very inconsistent
☐More consistent
☐Generally consistent
☐More consistent
☐Fully consistent

Learning interest

1. You like your math teacher.

☐Very inconsistent
☐More consistent
☐Generally consistent
☐More consistent

☐Fully consistent

2. You find math very interesting.

☐Very inconsistent

☐More consistent

☐Generally consistent

☐More consistent

☐Fully consistent
